# Repeated stage exposure reduces music performance anxiety

**DOI:** 10.3389/fpsyg.2023.1146405

**Published:** 2023-03-20

**Authors:** Victor Candia, Martin Kusserow, Oliver Margulies, Horst Hildebrandt

**Affiliations:** ^1^Department of Music, Institute for Music Research (IMR), Zurich University of the Arts (ZHdK), Zürich, Switzerland; ^2^Department of Information Technology and Electrical Engineering, Wearable Computing Lab ETH, Zürich, Switzerland; ^3^Swiss University Center for Music Physiology, Basel University of the Arts, Basel, Switzerland

**Keywords:** music performance anxiety, stage fright, coping, stage exposure, musicians’ medicine, musicians’ health, music physiology

## Abstract

**Background:**

High heart rate (HR) and restlessness are two important features of music performance anxiety (MPA). In a case report of a cellist suffering from this condition, we showed that HR and restlessness decreased after repeated live performances of the same musical excerpt, thereby positively modulating objective performance criteria and subjective components. Here, we largely replicate these results in a group of 18 string players reporting MPA.

**Methods:**

Objective measurement devices included a miniaturized electrocardiogram monitor and three 3-axis accelerometer loggers. Subjective measures included the Multidimensional Mental Health Questionnaire (MDBF) and a customized visual analogue scale (VAS) questionnaire for MPA. Non-artistic performance errors were assessed by music experts using a composite score for technical playing errors (i.e., intonation errors, omission of notes, and bowing noise). Data were collected from each study participant during three brief public solo performances of the same musical excerpt, with each performance occurring before a new audience on the same day.

**Results:**

From the 1st to the 3rd performance, HR, VAS, and playing error scores decreased significantly. MDBF (RU scale) showed a significant increase in calmness from the 1st to the 3rd performance on stage. HR and RU, VAS, and RU, as well as bow acceleration and overall duration of playing correlated significantly across participants and performances.

**Discussion and conclusion:**

We conclude that repeated stage exposure significantly reduces HR as well as restlessness and playing errors linked to MPA. Public performances are still successful when HR is significantly higher than during rest periods. These results underscore the importance of stage training to become accustomed to realistic public self-exposure. Musicians – especially students – should consider this component of stage training as an integral part of their practice routine. Therefore, stage training can reduce MPA, promote better live performances and prevent stress-related mental disorders and physical injuries. These result from excessive self-exercise strategies common in musicians experiencing MPA. HR monitoring should be an integral part of evaluating the effectiveness of interventions for better MPA management and efficient performance training.

## 1. Introduction

MPA is a form of anxiety that manifests in unwanted and unpleasant physiological and psychological responses. Increased sympathetic activity is a prominent indicator of MPA (Yoshie et al., 2009; DeCaro et al., 2011; Kenny et al., 2014; Kenny and Ackermann, 2015; Jane, 2022). In addition, effects of MPA on neuro-endocrine regulatory systems have been identified (Gomez et al., 2018; Haccoun et al., 2020).

Along with psychological factors, for instance, lack of general self-confidence, identified as a major source of MPA (Kenny et al., 2014), musicians list somatic signs caused by excessive physical arousal before or during performance among the main causes of their MPA. In addition, solo performance has major negative effects on MPA (Kenny et al., 2014). Negative post-event rumination has been reported to promote performance anxiety (Nielsen et al., 2018; Guyon et al., 2020) even beyond the stage (Haccoun et al., 2020). The factors mentioned above are not limited to orchestral musicians. Many players – whether of strings or not – suffer from MPA (see for example Yoshie et al., 2009).

Some knowledge on MPA management techniques have been reported. For instance, practical experience with training to minimize performance anxiety shows that building a personal stage choreography, including clapping, bowing or reverences, can promote a competent on-stage behavior, which also contributes to greater confidence when performing (Hildebrandt and Nübling, 2004; Hildebrandt, 2009; Williamon et al., 2014). Practicing under stress, i.e., off-stage, has also a positive effect on performance under real-life conditions (Oudejans and Pijpers, 2009, 2010). The use of video cameras leads to a strengthening of the individual’s self-perception and to a monitoring pressure that is typical for psychologically stressful situations (DeCaro et al., 2011; Papageorgi and Welch, 2020), therefore, it helps mimicking and practicing such situations.

Recent work has shown that not only the type of attention devoted to a motor task can improve or sustain skillful performance (Hildebrandt and Nübling, 2004; Mornell and Wulf, 2019) but also the timing at which an internal or external focus of attention is triggered. Using an internal focus of attention during performance preparation, then switching to an external focus of attention during performance, improves performance of a well-learned skill (Mornell and Wulf, 2019; Becker et al., 2020; Aiken and Becker, 2022) or at least is not detrimental to it (DeCaro et al., 2011). For example, DeCaro and colleagues have used distraction tasks designed to divert attention from an internal control focus while procedural tasks are performed under monitoring stress, and demonstrated their effectiveness (DeCaro et al., 2011). In other words, the focus of attention should be directed towards musical aspects, away from an error-oriented, analytical, and past-oriented focus. One could speak of a “musical memory training.” Such training involves so-called “semi-mental” training methods (i.e., true-to-the-original simulated instrumental and performance movements without an instrument, including body movements) designed to trigger mental representations of the target sound and musical performance (Hildebrandt, 2010) The goal of such training is to strengthen and stimulate the auditory-movement association – a very important safety donor on stage.

The importance of constructive feedback for a better management of MPA has ben also reported. In particular, the ability to devise solutions and strategies aimed at correcting weaknesses and working through them constructively (Hildebrandt and Nübling, 2004; Hildebrandt, 2009, 2010). The help of a constructively oriented audience to dampen the very common destructive review of undesirable outcomes (Nielsen et al., 2018; Studer et al., 2019; Guyon et al., 2020; Haccoun et al., 2020) that have been related to MPA, has been discussed to help construct a perspective for future performance (Brodsky, 1996, 1999; Hildebrandt, 2009).

Among other effects, sympathetic activation shortens cardiomyocyte action potentials, increasing HR (i.e., positive chronotropy) and shortening atrioventricular conduction (i.e., positive dromotropy; Schmidt et al., 2019). Parasympathetic activation reverses both (i.e., negative chronotropy; negative dromotropy; Schmidt et al., 2019). Thus, an important marker of increased sympathetic activity, and therefore of MPA, is a higher HR (Schmidt et al., 2019). Not surprisingly, several authors have included HR measurements in studies on for example stress during sports (Oudejans and Pijpers, 2009, 2010; Kusserow et al., 2010), repeated psychosocial stress induced by real and virtual stressors, including the same stressor over different periods of time (Schommer et al., 2003; Kothgassner et al., 2021, 2022), acute psychological stress (Trotman et al., 2019) and MPA (Yoshie et al., 2008; Yoshie et al., 2009; Kusserow et al., 2012; Kenny et al., 2013; Studer et al., 2014). In addition to HR, several authors have used heart rate variability (HRV) in the context of sports and exercise psychology to measure stress (Mosley and Laborde, 2022).

Experience can modulate mental and somatic responses mediated by sympathetic nervous system activity and thereby positively affects performance outcomes (Oudejans and Pijpers, 2009, 2010; Kusserow et al., 2012). Other authors have already demonstrated habituation of sympathetic responses to repeated psychosocial stress induced by real and virtual stressors (Kothgassner et al., 2021). Higher heart rates (i.e., higher sympathetic activity) have been shown to decrease significantly (i.e., lower sympathetic activity) after repeated exposure to the same stressor – in this study, a live audience – over different periods of time (Schommer et al., 2003; Kothgassner et al., 2021, 2022).

In this study, we tested the effects of experience on MPA levels using repeated live performances of the same musical excerpts in a group of string players who reported MPA. To our knowledge, apart from our previous case report (Kusserow et al., 2012), no similar study has been conducted. With the present study, we aimed to replicate the findings of the case report in a group of musicians affected by MPA. We aim to contribute to management techniques for MPA by reporting the effects of an intervention on momentary MPA (i.e., repeated live performances of the same musical excerpts within a day) and its objective and subjective evaluation. The prototypical set we tested is a key component of a successful stage training program conducted since 1998 at the Zurich and Basle Universities of Music (Hildebrandt, 2009, 2010).

To assess the effects of repeated live performance on the physiological and psychological components of MPA (i.e., stress desensitization) under comparable, real-world stress conditions, we created a performance situation in a highly demanding environment. It consisted in repeated solo performances in front of live professional audiences (Kenny et al., 2014; Papageorgi and Welch, 2020) differing between each performance. This added a dimension of implicit social appraisal identified as a key component of manipulating psychosocial stress and eliciting physiological stress reactivity (DeCaro et al., 2011). We measured physiological and psychological responses in string players, who represent one of the largest populations of musicians (Steinmetz et al., 2015) for whom we presented MPA monitoring data in the past (Kusserow et al., 2012).

To replicate a previous case report on MPA monitoring (Kusserow et al., 2012), we used HR as an objective indicator of MPA and assessed changes in HR before, during, and after the live performances. We correlated these changes with subjective assessments of MPA and with a standard measure of mood during three short public solo performances of the same musical excerpt, each on the same day and in front of a new audience. To monitor movement dynamics, we used accelerometers.

Evaluating HR alone to identify stress responses can be misleading because physical activity itself can act as a confounding variable. For this reason, we compared HR during performance and during play-free time by comparing HR while hopping in place for 60 s (baseline of maximal physical activity) with HR during performance. It needs to be stated that, in our previous case report (Kusserow et al., 2012), we compared exercise in daily life as recorded in a diary of activities performed during the day as exercise baseline. Therefore, maximal physical activity at baseline was better controlled in the study presented here, as all participants performed the same physical activity for the same amount of time.

In the past, technical and artistic components were used to evaluate the quality of a musical performance during MPA (Yoshie et al., 2009). The evaluation of artistic components, however, is difficult to reproduce. In this series of measurements, we therefore chose not to evaluate the artistic component. As in our previous case report (Kusserow et al., 2012), we used a manual, expert-based assessment of technical performance errors to determine the technical quality of the performance only. Technical components such as pitch (e.g., the correct note, note omissions) and rhythm can be better determined from the score and subjective impressions. For example, intonation quality has been shown to be a good predictor of listeners’ performance ratings (Johnson and Geringer, 2007), and some authors have shown that pitch errors are the easiest errors to notice (Doane, 1989; Waggoner, 2011).

We hypothesized that repeated stage exposure with the same musical excerpts would positively modulate MPA. Specifically, we hypothesized decreased HR, lower VAS scores for MPA, higher subjective well-being and calmness, fewer performance errors and, in addition, changes in bow movement dynamics from the first to the third performance.

## 2. Methods

### 2.1. Participants and ethics

The 18 string players (12 violin, 1 viola, 5 cello; 12 female, 6 male; mean age 21.11 SD 2.25; see Table 1 for comprehensive demographic data) gave their written informed consent prior to participation.

**Table 1:** Demographic data of participants.

Study Participants	Gender	Age	Instrument
1	Female	19	Violine
2	Female	20	Violine
3	Female	20	Violine
4	Female	20	Viola
5	Female	21	Violine
6	Female	21	Violine
7	Male	22	Cello
8	Male	19	Violine
9	Male	27	Violine
10	Female	22	Cello
11	Female	23	Violine
12	Female	21	Violine
13	Male	21	Cello
14	Male	19	Violine
15	Male	21	Cello
16	Female	23	Violine
17	Female	17	Cello
18	Female	24	Violine
	Mean	21.11	
	SD	2.25	

Requirements for participation in the study were an age between 18 and 35 years, no cardiac, respiratory, or metabolic problems, no diseases of the nervous system, including psychiatric diseases or diseases of the musculoskeletal system. No use of medications that affect the nervous system, cardiovascular system, or respiratory system, especially no use of beta-blockers. No health problems associated with the performance of athletic maneuvers such as jumping on the spot for 1 min (Table 2).

**Table 2:** Measurement methods and parameters.

Parameters measured	Methods
Objective measurement devices	
Heart rate (HR)	One lightweight (10 g) ECG-monitor (sampling rate = 256 Hz)
Body movement	Three 3-axis accelerometer loggers attached to: the outside of both forearms at the wrist – to measure movement during playing the left thigh above the knee – to measure total body movement (sampling rate = 32 Hz (range ± 4 g)
Subjective measurement tools	
MPA-level	10-item VAS (1 = no MPA, 10 = extreme MPA)
Level of current mental well-being	Multidimensional mental health questionnaire (MDBF); contains three bipolar dimensions of current mental well-being: good-bad mood (GS) alertness-fatigue (WM) and rest-restlessness (RU)

The study was conducted according to the guidelines of the Declaration of Helsinki^1^ for the treatment of experimental subjects. The local Ethical Committee at ETH Zurich approved the study protocol (EK 2010-N-57). The study was carried out at the Zurich University of the Arts (partner institution for this study). Participants received a monetary compensation of 30 Swiss Francs for their participation.

### 2.2. Objective data

A wearable system recorded cardiac activity and body movement simultaneously. The system consisted of an electrocardiogram (ECG) monitor (CamNtech, model: ActiwaveCardio, http://www.camntech.com/cntcardio.htm) and three 3-axis accelerometer loggers (Kusserow et al., 2010, 2012). The ECG monitor was a lightweight (10 g), single-channel waveform recorder with an integrated 3-axis accelerometer. It was attached to the chest of the musicians with two ECG electrodes (Ag/AgCl, 254mm^2^); the sampling rate was 256 Hz. The accelerometers were attached to the outside of both forearms at the wrist to measure movement during playing, and to the left thigh above the knee to measure total body movement at baseline (see below for more details). The sampling rate for the accelerometers was 32 Hz (range ± 4 g). The sensors were worn comfortably under the musicians’ clothing and did not interfere with their activities or performances (Kusserow et al., 2012). For a graphical representation of the multi-sensor system, see Figure 1 in Kusserow et al. (2012).

**Figure 1:**
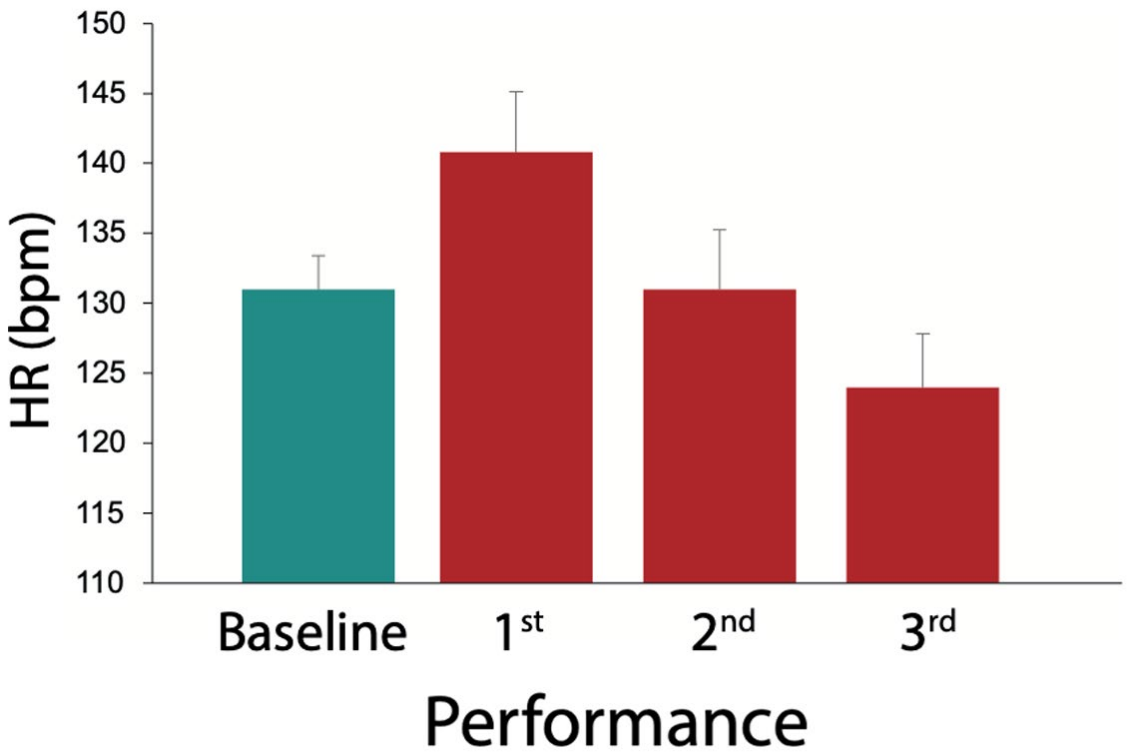
Heart rate (HR). HR changes from baseline to 3rd performance. Baseline (green) consisted of a 1 min run with hopping on the spot. Bars 2 to 4 (red) show HR during on-stage performance from 1st to 3rd performance. Shown are means and standard error (S.E.) of means.

All participants were recorded with the multi-sensor recording system, first at baseline, which consisted of a 60 s hopping on the spot after a standing quietly (baseline of maximal physical activity), and then during the live performance. The recording system remained attached to participants until their final performance on stage.

### 2.3. Subjective data

Before and after each performance, musicians were in the practice room to self-evaluate their live performance in terms of MPA level using a 10-item VAS (1 = no MPA, 10 = extreme MPA). In addition, they completed MDBF scales on the level of calmness and composure and on mood and alertness (Steyer et al., 1997).

We included the MDBF to control for current psychological well-being, i.e., well-being during measurements, because differences along the GS and WM scales would indicate changes in mood over time (i.e., more fatigue or low mood), both dimensions are known to influence experimental measurements in general. In addition, the MBDF contains a dimension that is closely related to psychological components associated with MPA, namely the degree of rest-restlessness. We did not include the Kenny Music Performance Anxiety Inventory because the questionnaire cannot provide information about current mood during – for instance – MPA episodes. In addition, to our knowledge, a validated German version of the K-MPAI was still pending by the time of measurements. The Multidimensional Mental Health Questionnaire (MDBF) consists of 24 items (each with a five-point response scale) to measure three bipolar dimensions of current mental well-being: good-bad mood (GS), alertness-fatigue (WM) and rest-restlessness (RU). All three scales can be divided into two parallel test halves each, which can be used to measure mental well-being over time. The internal consistency (Cronbach’s alpha) of the scales for the long form lies between α = 0.86 and α = 0.94, for the short forms between α = 0.73 and α = 0.89.^2^ Participants did not receive feedback on the quality of their performance.

### 2.4. Performance protocol

#### 2.4.1. Warm-Up

Before each performance, participants were not allowed to warm up for more than 3 min. To increase performance stress, participants were not allowed to warm up with passages they were going to perform on stage. About 2 min before the performance began, they left the practice room and walked over to the concert hall and onto the stage.

#### 2.4.2. On stage

All participants played their pieces from memory. During the performances, the violinists and the violist played in standing position.

#### 2.4.3. Audience and music pieces

A rotating audience of 15–20 people per performance, including professional-level music students and teachers, was present for all live performances. The musicians played the same piece of music in all three performances. Time interval between appearances on stage was about 1.5 h and were accompanied by a professional pianist when needed. Participants were asked to self-select the pieces they found most challenging and particularly well suited to trigger their MPA.

### 2.5. Analysis procedures

Audios and videos of the performances were recorded for later evaluation of the technical, non-artistic, quality of the performance. The beginning and end of each performance were determined by hand. Segmentation of data was performed using MATLAB software (MathWorks Inc., Natick, MA; http://www.mathworks.com/).

#### 2.5.1. Computation of HR and body motion

Heart beat series (RR-intervals) were computed using a free implementation^3^ of the Pan-Tompkins-Hamilton algorithm (Hamilton and Tompkins, 1986). To get a uniform sampling, heartbeat series were interpolated, matching the sampling of the corresponding body motion data (acceleration). The HR series $x_{HR}$ in beats⋅min^−1^ (bpm) was computed from the heartbeat time series $x_{RR}$ (in ms) by $x_{HR}=6\cdot 10^{4}\cdot x_{RR}{\;}^{-1}$. To obtain a representation for overall body motion dynamics, we computed the L2-Norm $||\left(a_{x},a_{y},a_{z}\right)||$ from the three axes of an acceleration sensor. To omit static acceleration components, the three acceleration axes were high-pass filtered (first order Butterworth filter, cut-off frequency 0.1 Hz).

#### 2.5.2. Computation of relative duration of execution

To assess the relative changes in playing time for each performer, we calculated the relative change in duration of execution by dividing the total duration of execution at the 3rd performance by the total duration of execution at the 1st performance. Therefore, values >1 indicated longer playing times during the 3rd performance compared with the 1st performance; values <1 indicated shorter playing times during the 3rd performance. Relative changes were presented as percentage differences.

#### 2.5.3. Assessment of non-artistic performance quality

Non-artistic performance errors were assessed by music experts using a composite score for technical playing errors: intonation errors, omission of notes, bowing noise).

Software developed in MATLAB was used to slice all complete audio samples into segments of equal length (3 s). The segmented audio samples were played to two professional musicians. Ratings were made by listening together to all individual excerpts from each performer for each of the three live performances. The experts collectively agreed on error categories and errors that fell into those categories for each segment. Only errors for which there was complete agreement among the evaluators were considered. The following categories of playing errors were considered: incorrect intonation, note errors (i.e., missing notes, rhythmic errors), and bowing errors (i.e., poor sound production such as bow whistles). Musical notes that had two or all three types of errors were assigned one error score per error category. We summed the number of technical playing errors from all three categories to obtain a composite score that represented an individual technical performance score for each performance (i.e., 1st, 2nd, and 3rd performance) and each player.

#### 2.5.4. Statistical analyses

Where appropriate, the Friedman Test, Wilcoxon Signed Rank Test, Spearman rank correlation, and Mann–Whitney U Test as well as Pearson’s *r* were used. For all comparisons, the significance level was set at *p* < 0.05. Our hypotheses would be effectively one-sided, considering that we tested based on our previous case report. Nevertheless, we report the two-sided test values here to avoid overestimating results; the one-sided *p*-value can be obtained by dividing the reported *p*-values by two.

### 2.6. Results

#### 2.6.1. Lower HR after repeated performances

The Friedman Test for changes in HR, including baseline (a 1 min run of hopping on the spot) and the three live performances, revealed highly significant changes (*p* = 0.00001). Individual *post-hoc* comparisons using the Wilcoxon Rank Test showed significant changes [HR during baseline < HR during 1st performance (*p* = 0.028); HR during baseline > HR during 3rd performance (*p* = 0.048); HR during 1st performance > HR during 2nd performance (*p* = 0.0002) and 3rd performance (*p* = 0.0002); HR during 2nd performance > HR during 3rd performance (*p* = 0.0005); see Figure 1].

#### 2.6.2. Subjectively less MPA after repeated performances

**Figure 2:**
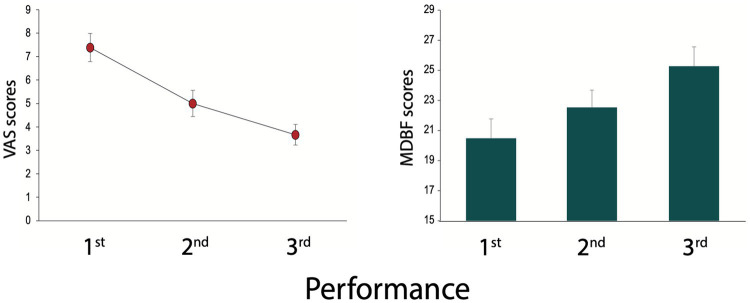
MPA-Questionnaire and MDBF-Questionnaire MDBF (RU scale). Tailored MPA questionnaire (**left**; VAS: 10 = MPA at maximum). MDBF (RU scale; **right**), which indicates the degree of rest-restlessness. Higher values mean more calmness and serenity. Shown are mean values and S.E. of the means.

The Friedman Test for the tailored MPA questionnaire for subjectively perceived performance anxiety (VAS: 10 = MPA at maximum) showed highly significant changes (*p* = 0.0009). Individual post-hoc comparisons using the Wilcoxon Rank Test revealed the following results: 1st performance scores >2nd performance scores (*p* = 0.001); 2nd performance scores >3rd performance scores (*p* = 0.0278); 1st performance scores >3rd performance scores (*p* = 0.00038; see Figure 2).

#### 2.6.3. Unchanged MDBF GS and WM scores

The Friedman Test for the good-bad mood (GS) and alertness-fatigue (WM) scales of the MDBF did not show significant changes.

#### 2.6.4. Higher MDBF RU scores from 1^st^ To 3^rd^ performance

The Friedman Test comprising the average of pre and post scores for all three performances for the RU scale of the MDBF (i.e., degree of rest-restlessness) revealed significant differences (*p* = 0.00637). Scores for the 1st compared to the 2nd assessment slightly failed to achieve significance (*p* = 0.0561). Higher scores of rest-restlessness were reported for the 3rd compared to the 1st assessment (*p* = 0.0164) and the 3rd compared to the 2nd assessment (*p* = 0.029). All individual comparisons were made using the Related-Samples Wilcoxon Signed Rank Test (see Figure 2).

#### 2.6.5. HR and RU correlated negatively across participants and performances

**Figure 3:**
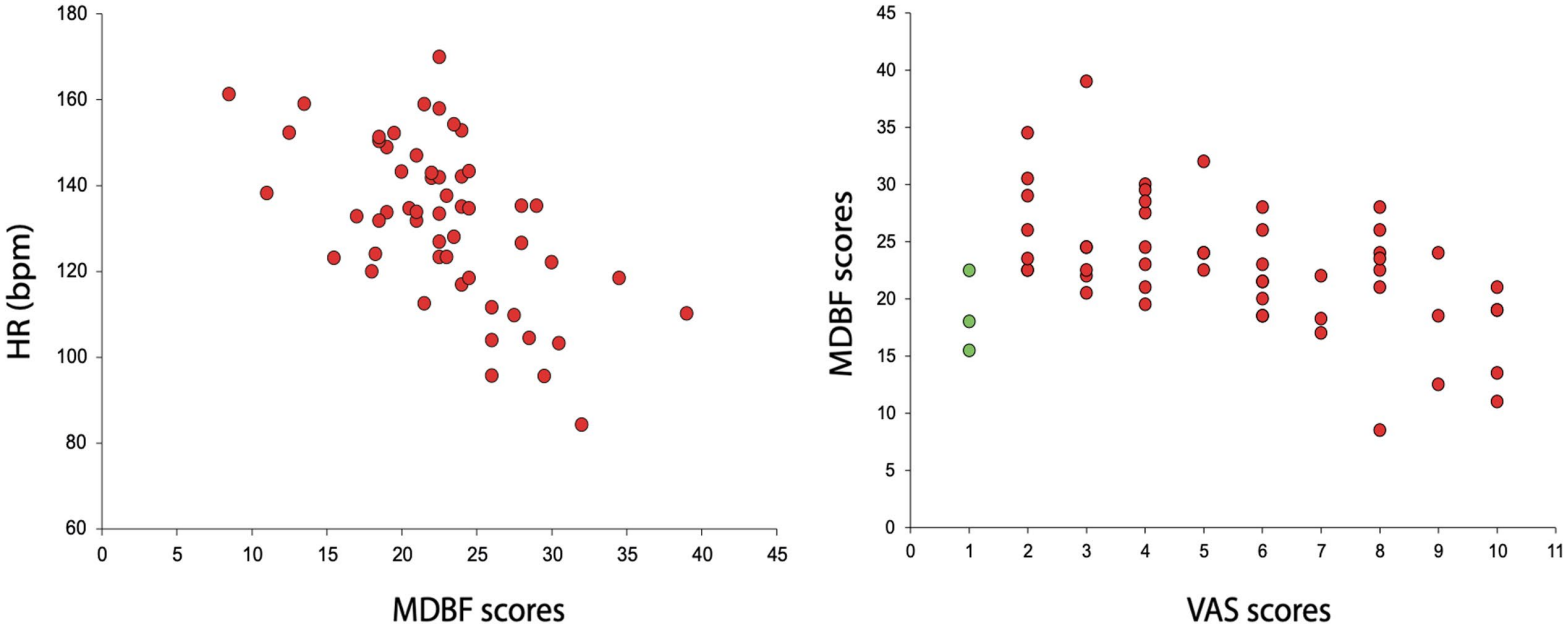
HR and RU correlation **(left)**, VAS and MDBF correlation **(right)**. Negative HR and RU Spearman’s rank correlation across participants and all three performances. Shown are all three RU scores and HR values for each participant **(left)**. Musicians with higher HR scores reported being less calm. Spearman’s rank correlation of negative VAS and RU **(right)** calculated across participants and all three performances. Shown are all three RU and VAS scores for each participant. The green dots represent the VAS scores of the only participant who scored all three performances with a 1.

There was a highly significant negative correlation between HR and RU scores (Spearman’s rank correlation across participants and performances (*r* = −0.54, *p* = 0.000030; see Figure 3).

#### 2.6.6. VAS and RU correlated negatively across participants and performances

There was a negative VAS and RU-MDBF correlation when calculated across all participants and all three performances (Spearman’s rank correlation *r* = −0.4 *p* = 0.00155). Note that one participant gave a VAS rating of 1 to all three performances while his HR pattern was like the one of the other participants; he could therefore be considered an outlier. A recalculation without this participant resulted in a Spearman’s rank correlation of *r* = −0.54 *p* = 0.00005; see Figure 3).

#### 2.6.7. Lower error scores after repeated performances

The total errors (i.e., intonation, note omissions, bowing errors) rated by two experts differed significantly among the three live performances (Friedman Test *p* < 0.00001). All individual comparisons using the Related-Samples Wilcoxon Signed Rank Test were significant: number of errors during 1st performance > number of errors during 2nd performance (*p* = 0.00036); number of errors during 2nd performance > number of errors during 3rd performance (*p* = 0.0018); number of errors during 1st performance > number of errors during 3rd performance (*p* = 0.0002; see Supplementary Figure 1).

#### 2.6.8. Two different patterns of overall duration of execution across performances

**Figure 4:**
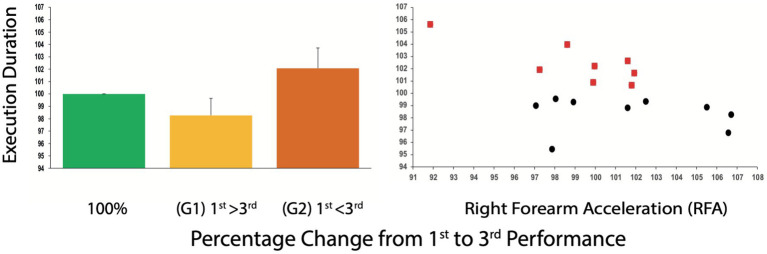
Changes in Overall Execution Duration **(left)** and Percentage Change in Right Forearm Acceleration (right), from 1st to 3rd performance. One group played faster (G1 n = 9) the other group (G2 n = 8) played slower during the 3rd performance **(left)**. 100% is shown for clarity, as a reference. On the right side, all participants split according to relative execution duration correlated to percentage changes in right forearm acceleration. Red squares represent G2 (longer execution duration at the 3rd performance).

Analysis of differences in duration of execution from the 1st to the 3rd performance, expressed as percentage changes, revealed two groups with opposite patterns of overall playing speed (Mann–Whitney U, two-sided test. U value = 0, critical value of U at *p* < 0.05 = 15, *p* = 0.00044): One group (G1 *n* = 9) played faster during the 3rd performance, whereas the other group (G2 *n* = 8) played slower. One participant from G1 was excluded because her playing time was artificially prolonged due to memory lapses, which made the 1st performance slower. Note that including this participant would have made the differences even stronger; see Figure 4).

#### 2.6.9. HR split by duration of execution was similar between subgroups

Percentage changes in HR from 1st to 3rd performance did not differ between G1 and G2 subgroups (Mann–Whitney U value = 23, critical value of U at *p* < 0.05 = 15, *p* > 0.05), nor did they differ in baseline HR (Mann–Whitney U: U value = 35, critical value of U at *p* < 0.05 = 15 *p* > 0.05). Individual comparisons between subgroups and corresponding performances (e.g., G1, 1st performance vs. G2, 1st performance) were not significant (Mann–Whitney U, *p* > 0.05, for all three comparisons). Friedman Tests for G1 and G2 separately, were both significant (G1 *p* = 0.0003; G2 *p* = 0.00034). Individual post-hoc comparisons using the Sign Test for both groups separately yielded the following results: G1 1st > 2nd *z*-value = 3, *p* = 0.0027; 2nd > 3rd performance: *z*-value = 2.33, *p* = 0.01963; 1st > 3rd: *z*-value = 3, *p* = 0.0027. G2 1st > 2nd: *z*-value = 2.83, *p* = 0.00468; 2nd > 3rd performance: *z*-value = 2.282, *p* = 0.00468; 1st > 3rd: *z*-value = 2.83, *p* = 0.00468 (note that the reduced number of n-participants per group did not allow calculation of an exact *p*-value using the Related-Samples Wilcoxon Signed Rank Test; see Figure 4).

#### 2.6.10. Uncorrelated percentage changes in RFA and HR

Percentage changes in right forearm (i.e., bow’s arm) acceleration (RFA) were not correlated with percentage changes in HR (*r* = −0.0008, *p* > 0.05). Percentage changes in duration of execution were also not correlated with percentage changes in HR (*r* = −0.32, *p* > 0.05). This was also true for both subgroups.

#### 2.6.11. Moderate and negative correlations for percentage changes in duration of execution and percentage changes in RFA-values

The Pearson’s r for the correlation of the percentage changes in duration of execution with the percentage changes in RFA values was −0.51 (*p* = 0.036484), indicating a moderately negative relationship, with bow acceleration accounting for 26% of the changes in duration of execution from first to 3^rd^ performance (see Figure 4).

Percentage changes in HR did not correlate with percentage changes in RFA (*r* = −0.0008, *p* > 0.05). This was also true for both subgroups.

## 3. Discussion

### 3.1. Overview of the obtained results

In this study, we showed that repeated live performances with the same musical excerpts lowered HR, led to less subjective MPA and more calmness among participants. HR and level of calmness were negatively correlated: the higher the measured HR, the less calmness among participants. In addition, repeated live performances resulted in lower error scores, most notably, without additional off-stage practice. Analysis of differences in duration of execution from the 1st to the 3rd performance revealed two groups with opposite patterns of overall playing speed: one group played faster at the 3rd performance; the other group played slower. The groups did not differ in HR from 1st to 3rd performance, nor did they differ in baseline-HR or individual comparison of the three different time points. When analyzed separately, both subgroups showed similar HR patterns to all participants when considered as a single group. Percentage changes in RFA were moderately and negatively correlated with percentage changes in duration of execution, with bow acceleration explaining 26% of the changes in duration of execution from the 1st to the 3rd performance. It is noteworthy that percentage changes in RFA and in duration of execution did not correlate with percentage changes in HR. This was also true for both subgroups.

#### 3.1.1. HR physical versus mental components

The patterns of HR across live performances cannot be explained by physical activity alone. For instance, physical activity reached its maximum during baseline (i.e., hopping on the spot for 1 min), and not during performances on stage. The patterns of HR changes were also consistent even when the data were analyzed based on the different patterns of duration of execution. Our data confirms once more that experience can modulate mental and somatic responses mediated by sympathetic nervous system activity positively affecting performance outcomes (Oudejans and Pijpers, 2009, 2010; Kusserow et al., 2012). It also confirms the habituation of sympathetic responses to repeated psychosocial stress induced by real and virtual stressors (Kothgassner et al., 2021), with heart rates significantly decreasing after repeated exposure to the same stressor over different periods of time (Schommer et al., 2003; Kothgassner et al., 2021, 2022).

Because the percentage difference in RFA in both subgroups from 1st to 3rd third performance was minimal (+1.6% for G1 and −0.07% for G2), the changes in RFA cannot explain the observed changes in HR from 1st to 3rd performance.

#### 3.1.2. Baseline

An appropriately elevated heart rate - i.e., under physiological stress conditions such as running - is appropriate to the challenge of the environment and can be discharged by the large striated muscle groups. Similar arousal during a musical performance would be less appropriate because it leads to undesirable physiological responses such as tremors, arch tremors, restlessness as non-functional ways to discharge the excessive sympathetic arousal. Nevertheless, there were no complains on such non-functional discharges of excessive sympathetic arousal and no such signs were evident during evaluation of errors in the selected excerpts. For example, bow errors accounted for 18, 20, and 23 percent of the errors during the 1st, 2nd, and 3rd performances, respectively. In the case of adverse physiological responses, one would expect a higher number of for example bow errors during the 1st performance because this performance was closer to baseline, the only time when jumping on the spot was introduced. Because percentages of bow errors remained fairly unchanged across performances, we conclude that the physical challenge we used as a baseline did not interfere with the results.

#### 3.1.3. Sympathetic reactivity over the three performances

Time interval between appearances on stage was about 1.5 h, during which no practice or passage corrections with the instrument were allowed. Therefore, we cannot completely rule out the possibility that the decreasing sympathetic reactivity across the three performances was due in part to the depletion of adrenaline, simply because the performances occurred in close succession. This would indeed be a general effect one would expect after repeated performances on stage. Nevertheless, we are convinced that the repeated performance of the same piece of music in front of a changing audience played a significant role in the marked changes in HR. In other MPA investigations performed on 1 day those who had previously performed a Debussy piece had significantly lower HR (Kenny et al., 2013). However, it could also be that both the repeated performance on stage and the repeated performance of the same pieces in front of an audience had a combined, and strong desensitizing effect.

A similar study with performances on consecutive days or a few days apart with the same and different repertoires could provide new insights but would introduce a confounding factor that we neutralized here, namely more practice hours between performances. In the present study, we could rule out the results being explained by more physical training, a strategy reportedly used to cope with MPA, for which our results offer an alternative.

In future studies, resting HR could also be measured in time-lapse, including a few days before performance. For example, this would reveal HR profiles as the day of performance approaches and hours and days after performance. The information could be correlated with validated measures of state anxiety, fear of negative evaluation or amount of MPA.

#### 3.1.4. Changes in RFA

Since the changes in RFA were not correlated with HR and there was no opportunity to practice between the three performances, perhaps they show the different degree of confidence in the performed pieces due to an on-stage practice effect.

#### 3.1.5. Mental components as a function of the MPA level

In total, body acceleration remained unchanged from the first to the third performance, thus it cannot explain the observed changes in HR. We conclude that HR changes, as observed here, most likely depend on changes in mental components as a function of the MPA level.

In the present work, as in our previous work (Kusserow et al., 2012), we were not interested in clarifying exactly which mental components are responsible for the changes in HR. In fact, we consider it one of the contributions of both studies to show that it is possible to separate physical and mental components contributing to the observed HR during MPA episodes. The amount of physical exercise during performance cannot explain the observed changes in HR (especially during the first performance), so we conclude that mental factors are responsible. This conclusion is entirely in line with previous work examining HR responses during practice and training compared to HR responses under evaluation and competition conditions when the same tasks – with the same physical content – were performed (Yoshie et al., 2008, 2009; Kusserow et al., 2010). Future research could shed light on specific mental components of MPA and HR, such as whether subjects with high fear of negative evaluation (FNE) show particularly high HR responses compared to MPA subjects with low FNE when measured over time following an MPA management intervention. This seems plausible, as athletes with high FNE showed a significant increase in anxiety associated with lower performance in stressful situations (Mesagno et al., 2012), and anxiety and HR have been shown to be higher during evaluation and stress conditions (Noteboom et al., 2001; Yoshie et al., 2008, 2009; Kusserow et al., 2010, 2012). We can only speculate about what mental elements are at work in this series, as they have not been the focus of our attention. Some or all participants in our group may have had negative thoughts during performance, especially during the first performance. This is related to the mental components of anxiety, such as in testing situations, also known as cognitive performance anxiety or CPA. Recently, it has been shown that individuals with high trait CPA have higher anxiety levels and attention to negative thoughts (Angelidis et al., 2019), which can have a negative impact on performance. In addition, musicians instructed to use an internal attentional focus during performance performed worse on the technical and musical aspects of their performances than those instructed to use an external attentional focus (Mornell and Wulf, 2019). Although we did not ask about the thoughts participants had during performance, the lower number of errors, higher MDBF scores, lower MPA scores on the VAS, and lower HR from the first to the third performance suggest that if affected, participants were less affected by the two performance-impairing cognitions mentioned above from performance to performance.

#### 3.1.6. Somatic and cognitive manifestations

Future work should consider that somatic and cognitive manifestations are at least partially independent of each other – some have mainly somatic anxiety, others mainly cognitive, others a combination of both. In the latter group, somatic manifestations may be high, cognitive low, or vice versa. They may also have one or the other manifestation, depending on how close the performance is and the type of performance (e.g., “stake”). Each of these subsets of MPA manifestations, if adequately determined, may show a different pattern of HR responses. However, assuming that in our sample there might be one or more representatives of one of the groups with different somatic and cognitive manifestations of anxiety, the consistency of HR profiles across participants in this series, in our previous case report (Kusserow et al., 2012), and in reports on sports competitions (Kusserow et al., 2010) suggest(s) that HR responses are less vulnerable to somatic and cognitive manifestation of MPA. Moreover, our assumptions are consistent with other studies in the field of music performance. Previous data on physiological responses of music students on stage, particularly data on cardiac and respiratory responses, already revealed comparable physiological responses in those with high subjective ratings of anxiety and those who rated themselves as having low anxiety on stage (Studer et al., 2011a, 2012, 2014). Finally, the heterogeneous group we studied in the context of MPA interventions is most likely representative of the type of populations one would encounter in a Western music high school where musicians are trained with classical virtuoso repertoire – underscoring the general relevance of our findings.

#### 3.1.7. Duration of execution

Trait differences have been considered in the past in the context of MPA, for example, when examining predictors of MPA during skilled performance of musical pieces (Kenny et al., 2013). Such underlying differences may be evident not only in psychometric measures but also in physical parameters. For instance, consequences of stress are freezing and the limiting of movement amplitudes (Higuchi et al., 2002), increased arm stiffness and decreased force regulation during arpeggio performance in skilled pianists (Yoshie et al., 2008), and less well-controlled force during a pinch grip (Noteboom et al., 2001) – the latter being closely related to the performance of left and right hand movements in string instruments. In our previous case report, we also found that 3rd performance was faster in comparison to first performance (Kusserow et al., 2012). Therefore, we expected that the analysis of variations in duration of performance would reveal some differences in movement profiles related to MPA. Although the small number of participants precludes solid conclusions, the variable ‘duration of execution’ revealed two distinct subgroups in the collective measured here. The one subgroup showed too much drive to move (i.e., it was unfrozen and played faster at first performance), whereas the other showed the opposite pattern (i.e., it was frozen and played slower). Thus, these movement responses may represent two distinct and innate responses to dysregulated arousal as caused by anxiety and should be considered in future research aimed at implementing better management strategies for MPA (i.e., some individuals may need to be energized while others may need to be calmed to a medium level of arousal prior to performance).

#### 3.1.8. HR and subjective MPA scores

The participants’ subjective impression of their MPA and calmness scores largely mirrored HR patterns. In addition, VAS and MDBF scores were negatively correlated across participants and performances, highlighting the strong agreement between the subjective data. Furthermore, the negative correlation between HR and RU-MDBF across all participants and all three performances rounded out the analyses, showing congruency between physiological measures and subjective data.

Overall, these results are largely consistent with our previous case report (Kusserow et al., 2012) and clearly separate physical from mental components triggering HR responses during MPA episodes. Therefore, we consider HR patterns as key parameters in the investigation and evaluation of MPA, for instance, MPA management interventions.

There are some limitations to some of the methods we used. For instance, the subjective data collected relied on individual participants’ understanding of MPA. Therefore, we did not define MPA for each participant before asking them to rate their own MPA. In addition, the questionnaire did not include questions about physiological symptoms such as hand sweating or muscle stiffness, two important symptoms indicative of higher MPA. These limitations arose in part from the fact that we wanted to replicate previous findings reported in a single case report (see (Kusserow et al., 2012). Certainly, a unidimensional VAS alone would be not adequate to assess the complex multi-factorial nature of MPA. For this reason, we correlated VAS and MBDF to in part limit this shortcoming. In other research on MPA in experienced musicians, some authors have also used 10-point scales in conjunction with other measures to have participants rate their level of pre-performance nervousness (Kenny et al., 2013). In this series, as in our previous case report (Kusserow et al., 2012), we avoided making explicit references to physiological symptoms and commenting on performance quality because any explicit mention would risk triggering an internal attentional focus (Mornell and Wulf, 2019), and fear of negative evaluation (Watson and Friend, 1969), both of which have the potential to increase anxiety (Kenny et al., 2013; Mornell and Wulf, 2019), and therefore affect measured parameters across performances (i.e., over time). Future research should incorporate other validated questionnaires to accurately measure immediate anxiety. For example, the Immediate Anxiety Measures Scale (IAMS) provides valid and reliable cognitive and somatic anxiety scores and can be used to measure task-specific anxiety. It has been correlated with HR reactivity measured in a single session (Trotman et al., 2019). In addition, the certified German translation of the K-MPAI – R could be used (Kenny, 2017) as all translated versions appear to have adequate reliability to measure anxiety levels (Antonini Philippe et al., 2022).

Future research should incorporate trait measures, measures of overall MPA (e.g., K-MPAI) and/or a measure of self-efficacy and/or depression. These measures could be then correlated with objective measures such as HR, or to changes in MPA over much longer periods of time to validate changes after longer periods of intervention.

#### 3.1.9. The proper context to manage against MPA

We believe that the context we created is well suited for studying and training how to deal with MPA. We created a solo-performance environment directly comparable with real audition settings, which also contained several social-evaluative factors. These have been reported to be major triggers of explicit monitoring, a form of disruptive and excessive attention to skill processes and procedures during the performance of well-learned, complex motor tasks (DeCaro et al., 2011; Guyon et al., 2022; Sokoli et al., 2022). For instance, the audience included professional musicians and advanced music students, and appearances were videotaped. The data obtained, especially the HR, show that the participants considered the situation as a real performance consistent with performance under pressure (Baumeister, 1984). Some of the effects observed here have also been observed in other performances under pressure: For instance, high levels of performance anxiety were indicated by higher HR activity on the day of the concert (Hildebrandt et al., 2012), in solo performances with direct or indirect assessment (Yoshie et al., 2009; Hildebrandt et al., 2012; Kusserow et al., 2012; Papageorgi and Welch, 2020), and in sports (Kusserow et al., 2010). We also increased stress by preventing participants from rehearsing or warming up with the excerpts they were about to perform (even between performances). The measurement situation was thus clearly a demanding one (DeCaro et al., 2011; Papageorgi and Welch, 2020).

#### 3.1.10. Selection of pieces

One possible limitation of the study is that there was no prior measurement of technical mastery, nor was there any measure of familiarity or prior performance history of the excerpts selected. This is worth noting because some authors have shown that experience with test pieces can affect muscle tension and heart rate (Kenny et al., 2013). Musicians were asked to select a musical excerpt that, in their personal experience, would trigger their MPA. For some musicians, even technically less difficult pieces may trigger MPA if there was additional underlying psychopathology, which we did not completely control for (see Methods section for the inclusion and exclusion criteria). However, even in the absence of psychopathology, the degree of technical difficulty and the evaluation of that difficulty are subjective: neither a perfect nor an imperfect rendition (musically and technically speaking) would indicate that MPA has or has been not triggered. This was an important reason why we asked the musicians to self-select the pieces that triggered their MPA and why we compared each set of three performances individually (i.e., each performance of each person was compared with itself, i.e., three renditions of the same excerpt in a time distance of about 1.5 h). In contrast to our report, Kenny et al. compared changes in physiological and subjective ratings when different musical excerpts were performed in a laboratory session. Notably, those who had previously performed a Debussy piece had significantly lower HR (Kenny et al., 2013). This finding is consistent with one of the main findings of the present report, that experience with the same piece on stage correlated with lower HR from the first to the third live performance. In addition, we used a sum score – not an average score – for the error evaluation to in part control for the fact that the selected pieces were not comparable. An alternative and more homogeneous selection criterion for the choice of the evaluated pieces could be achieved if the measurements were performed, for example, during a performance competition were excerpts with similar difficulty are performed. Such a measurement situation has been deployed by others in the past to assess MPA in pianists (Yoshie et al., 2009). Within the context of sports, performing seminal work on stress arousal measurement during ski jumping, Kusserow et al., showed that HR significantly increased from training to qualification to Olympic competition (Kusserow et al., 2010). The HR values observed during competition were comparable to the values we observed here, confirming that the evaluative context of performance more than a specific set of tasks have the power to trigger MPA.

#### 3.1.11. The number of errors

The number of errors decreased from the 1st to the 3rd performance without additional off-stage rehearsals. These results replicate the findings of our previous case report (Kusserow et al., 2012). While one musician stopped playing during one of the performances, all others performed without breakdowns. This confirms that performance is possible even under very stressful conditions and that the participants had most likely practiced enough to play by heart in front of an audience, with a comparable technical level. A possible limitation of our study is that we did not ask participants about their thoughts during execution. This information could provide clues to the reasons and nature of the errors they committed. While we avoided giving participants any instructions throughout the measurements, it is reasonable to assume that they instructed themselves in some way to cope with their anxiety. Recent work on so-called ironic errors in reactive motor performance under pressure has shown that the type of instruction can modulate such errors, at least in reactive motor tasks (Gorgulu et al., 2019) that are somewhat different to the kind of motor task performed here. In the domain of musical performance, Mornell and Wulf have shown that explicitly instructing performers to adopt an external attentional focus leads to significantly greater technical precision during performance under pressure (Mornell and Wulf, 2019). It is conceivable that a reduction in anxiety level allowed musicians to unconsciously shift from an internal to an external focus of attention from 1^st^ to 3^rd^ performance. Therefore, interventions to address MPA could include both elements: repeated live performances of the same musical excerpts along with explicit instructions to potentiate external foci of attention (Hildebrandt and Nübling, 2004; Mornell and Wulf, 2019). Future research with comparable study designs to the one used here could include a more in-depth error analysis to develop a set of appropriate instructions that could counteract unintentional errors even under anxiety conditions while performing.

### 3.2. Contributions

Our data can contribute to understand some aspects of MPA management. The causes of MPA can be multifactorial and diverse (Brodsky, 1996; DeCaro et al., 2011; Studer et al., 2011b; Kenny et al., 2014; Kenny and Ackermann, 2015; Haccoun et al., 2020; Papageorgi and Welch, 2020; Jane, 2022; Sokoli et al., 2022). Therefore, our data can only partially contribute to the management of MPA. In particular, the components related to the modulation of physiological parameters strongly associated with MPA may be affected by desensitization. For instance, practicing under stress is clearly beneficial for reducing HR (Oudejans and Pijpers, 2009, 2010; Kusserow et al., 2010, 2012). Among the causes of MPA, excessive physical arousal before or during performance was the second most frequently cited cause in previous reports, accounting for 78.3% of participants’ responses (Kenny et al., 2014). Obviously, the reduction of sympathetic activity had a positive effect on the quality of the performance – as measured in our study by the smaller number of errors from the 1st to the 3rd performance – and the subjective evaluation of the situation – as measured by the lower VAS-MPA values and the greater calmness. Therefore, particular attention should be paid to sympathetic responses during performance in studies on MPA. This is also important because high sympathetic activation during training has been reported to lead to better performance in real-life situations (Oudejans and Pijpers, 2009) and should therefore be an important goal of any training for MPA management.

### 3.3. Future investigations

We have presented here data on the effects of repeated live performances of the same musical excerpts within a short time frame of about 4 h. However, multiple elements should also be considered when searching for appropriate training strategies to manage MPA. Therefore, several other training elements should be investigated in further research, and one of the tools to assess their effectiveness should consider over-time changes in HR. For instance, lack of general self-confidence was identified as a major source of MPA (Kenny et al., 2014), and our practical experience shows that building a personal stage choreography, including clapping, bowing or reverences (Hildebrandt and Nübling, 2004; Hildebrandt, 2009), contributes to greater confidence when performing. An alternative or complement to HR could be the use of HRV to assess changes induced by, for example, MPA interventions for self-regulation. However, their complexity and interpretation should not be underestimated, as there are several theories that make predictions about the role of vagal activity. In addition, there are limitations to measuring stress with HRV, and the relationships between subjective ratings and HRV are inconsistent and difficult to interpret, as are many of the numerous parameters that have been assessed with HRV. Nevertheless, studies of vagally mediated HRV are promising (Mosley and Laborde, 2022), and could also be performed as part of future investigations on MPA management techniques.

#### 3.3.1. Psychological interventions: Positive psychology and cognitive behavioral therapy

Negative post-event rumination has been reported to promote performance anxiety (Nielsen et al., 2018; Guyon et al., 2020) even beyond the stage (Haccoun et al., 2020).

The use of Multi-component Positive Psychology interventions (MPPIs) in MPA could be considered. Randomized control trials have shown that they can have a positive effect on anxiety and stress, although more well-controlled data are needed (Hendriks et al., 2020).

The use of cognitive behavioral therapy (CBT), can consider multiple aspects involved in the causation of MPA, even in individuals who experience MPA but do not suffer, for example, from generalized anxiety disorder (GAD). This is particularly noteworthy because professional orchestral musicians consider ‘pressure from self’ to be the cause of their MPA (Kenny et al., 2014), and because it has recently been shown that musicians’ MPA is strongly predicted by GAD – and not social anxiety disorder (SAD; Wiedemann et al., 2022). CBT has been shown to be particularly effective for treating GAD, to the same extent as medication (Otte, 2011) and with long-lasting effects (Bandelow et al., 2018). Again, a pre-to-post intervention evaluation of the efficacy of such psychological interventions by means of physiological and psychological tests and their correlation is needed. This appears feasible, as we have prototypically shown here for repeated live performances under pressure.

Thus, negative post-event ruminators may benefit from the use of explicit strategies elaborated with positive psychology and cognitive behavioral therapy and they should be also evaluated in relation to physiological reactions, pre- and post-interventions. In the same line of constructive interventions, the training of a constructively oriented attention focus needs similar evaluations (Brodsky, 1996; Hildebrandt and Nübling, 2004).

### 3.4. Conclusion

Stage training reduces both HR and restlessness during MPA. Successful public performances under higher HR than HR during rest periods remain possible. These results underscore the importance of practicing on stage to get used to it, not just practicing off stage. Musicians – especially students – might consider this type of training a necessary step towards better managing MPA. Such training can contribute to better live performances and prevent destructive mental attitudes and physical injuries from over-practicing. HR monitoring should be an integral part of evaluating the effectiveness of interventions to better manage physical activity and properly train performance at higher HR (i.e., high sympathetic activation), especially considering that wearable technology and devices such as wristwatches equipped with the necessary technology are readily available. Moreover, textile-integrated sensos could be also considered. The long-term effects of the kind of intervention presented here (i.e., over days, weeks and even months post intervention) remain to be investigated. Following our earlier report, we used three performances in 1 day here. Questions arise about how many consecutive performances and what time interval is the optimal training protocol to achieve and maintain acclimatization. Other questions relate to the use of HR data for training and how it should be presented and applied (e.g., on-site or during visualization). Still other questions are possible and need to be investigated.

## Data availability statement

The original contributions presented in the study are included in the article/Supplementary material, further inquiries can be directed to the corresponding author.

## Ethics statement

The studies involving human participants were reviewed and approved by the local Ethical Committee at ETH Zurich (EK 2010-N-57). The participants provided their written informed consent to participate in this study.

## Author contributions

VC planned the study, collected and analyzed data, performed statistical calculations on all data, and wrote the manuscript. MK planned the study, collected and analyzed sensor data, critically reviewed the manuscript, and contributed to the discussion. OM collected audio and video data, analyzed subjective data, and critically reviewed the manuscript. HH planned the study, recruited participants, analyzed data, and wrote the manuscript. All authors contributed to the article and approved the submitted version.

## Funding

This work was supported in part by a grant of the Swiss National Science Foundation to HH and VC (K-13 K1-120706).

## Conflict of interest

The authors declare that the research was conducted in the absence of any commercial or financial relationships that could be construed as a potential conflict of interest.

## Publisher’s note

All claims expressed in this article are solely those of the authors and do not necessarily represent those of their affiliated organizations, or those of the publisher, the editors and the reviewers. Any product that may be evaluated in this article, or claim that may be made by its manufacturer, is not guaranteed or endorsed by the publisher.

## Footnotes

^1^ http://www.wma.net

^2^ https://www.testzentrale.ch/shop/der-mehrdimensionale-befindlichkeitsfragebogen.html#1+1

^3^ http://www.eplimited.com
