# Supplementary material for: Repeated stage exposure reduces music performance anxiety
Source: Front Psychol. 2023 Mar 20;14:1146405. doi: 10.3389/fpsyg.2023.1146405 (PMC10067860; doi:10.3389/fpsyg.2023.1146405)
Supplement: Supplementary file 1 [file Data_Sheet_1.docx]

Supplementary Material

**Repeated Stage Exposure Reduces Music Performance Anxiety**

Victor Candia^*^, Martin Kusserow, Oliver Margulies, Horst Hildebrandt

*** Correspondence:** Corresponding Author: [victor.candia@zhdk.ch](mailto:victor.candia@zhdk.ch)

## Supplementary Figures

**Supplementary Figure 1.** Sum of the performance errors (intonation, note omissions, bowing errors) evaluated by two experts. Only errors where there was complete agreement between the experts were considered.
